# Characteristics of corneal donors from local procurement at Lions Eye Bank Jakarta: an eight-year report

**DOI:** 10.3389/fmed.2026.1862074

**Published:** 2026-08-17

**Authors:** Lily Silva Ardiani, Rommi Aderiyansah, Sharita Rosalyne Siregar

**Affiliations:** 1 Lions Eye Bank Jakarta, Jakarta, Indonesia; 2 JEC Eye Hospitals and Clinics, Jakarta, Indonesia; 3 The Association of Eye Banks of Asia (AEBA), Singapore, Singapore

**Keywords:** characteristics, cornea, corneal tissue, donor profile, local procurement

## Abstract

**Purpose:**

To present corneal donor demographics obtained from local procurement at Lions Eye Bank Jakarta in Indonesia.

**Methods:**

This single-center retrospective study was conducted from March 2018 to February 2026. Donor characteristics were collected. Corneal tissue grading, based on endothelial cell density (ECD), was performed using the EBAA standard.

**Results:**

A total of 47 donors (93 corneal tissues) were included in the final analysis, with mean age of 64.60 years (SD 19.83; range 10–100). The majority age group was >65 years (25, 53.2%), with the male predominance (24, 51.1%). Cardiac arrest (13, 27.7%) was the leading cause of death. Most procurements were performed at funeral homes (32, 68.1%). Grade A (84, 90.3%) corneal tissues dominated our cohort, with a mean ECD of 2,270.03 cells/mm^2^ (SD 308.36; range 1,418–2,985). Penetrating keratoplasty (39, 41.5%) and DSAEK (33, 35.1%) were the most common surgeries, with a mean preservation-to-surgery time of 9.25 days (SD 2.8; range 1–14). Using multivariable binary logistic regression, no significant associations were observed between donor age, gender, and preservation solution and ECD grade or utilization. Only procurement time predicted ECD grade (OR 0.999; 95% CI 0.998 to 1.000; *P* = 0.025)

**Conclusions:**

Male, older age, and cardiac arrest were the most common donor profiles. Grade A corneal tissues predominated, and penetrating keratoplasty was the most common procedure. Donor age, gender, and preservation solution were not associated with ECD grade or corneal utilization; only procurement time was. Longer procurement time after death was associated with lower odds of obtaining Grade A corneal tissue.

## 1 Introduction

Corneal opacity remains one of the leading causes of blindness worldwide, with more than 5.5 million people are bilaterally blind or having moderate to severe visual impairment, and more than six million people are unilaterally blind due to corneal opacities (1, 2), including in Southeast Asia and Indonesia (3, 4), An estimated 34.9 million people in Indonesia had vision loss, and 3.73 million were reported blind (5). According to Tran et al. (6) the incidence of corneal disease in Indonesia resulting in severe visual impairment and blindness is around 0.28 (confidence interval, 0.08–0.048). Compared to other Asian countries, Indonesia still has a high rate of corneal blindness, which requires better addressing and comprehensive treatment to diminish the burden of corneal disease.

The 2016 Global Survey on Corneal Transplantation and Eye Banking reported that 185,000 corneal transplants were performed in 116 countries, and approximately 284,000 corneal donors were obtained in 82 countries (7). However, the gap between the demand for corneal transplants and the supply of corneal tissue persists. More than 12.7 million patients remain on the corneal transplant waiting list, and over half of the corneal tissue was procured by the USA and India (7). Indonesia was classified as “embryonic” in the 2016 Global Survey on Corneal Transplantation and Eye Banking, indicating that while corneal transplantation is available and there is at least a small facility for corneal preservation, the number of corneal tissues procured is extremely limited (7). Until recently, Lions Eye Bank Jakarta (LEBJ) conducted a 2024 study analyzing keratoplasty trends over 6 years at one of the tertiary eye hospitals in Jakarta, Indonesia (8). These findings suggest that Indonesia has progressed beyond the embryonic stage as described in the previous 2016 Global Survey, although a national registry remains unavailable.

LEBJ is a not-for-profit organization that procures, evaluates, and distributes corneal tissue for transplantation across Indonesia (9). LEBJ was established in 2018 and is affiliated with Bank Mata Indonesia (Indonesian Eye Bank). Since 2018, LEBJ has also been affiliated with SightLife USA, a not-for-profit organization focused on eliminating corneal blindness in the USA and developing countries. LEBJ joined the Association of Eye Bank of Asia (AEBA) in 2020 and the Global Alliance of Eye Bank Associations in 2024. All the procedures in LEBJ follow the AEBA and the Eye Bank Association of America (EBAA) medical standards. Recently, in December 2025, LEBJ was certified by the Ministry of Health of the Republic of Indonesia for eye bank standardization. We are the first eye bank in Indonesia to receive this standardization certification.

The success rate of corneal transplantation is influenced by donor and recipient selection, preparation of corneal tissue, keratoplasty technique, and postoperative management (10, 11). High-quality corneal procurement and sufficient eye banking also contributed to the success of corneal transplantation. Corneal procurement in Indonesia faces many challenges, including a very limited number of voluntary corneal donors and eye banks, various religious beliefs, myths, and misconceptions about corneal donors, and donor consent from the donor's family. Tran et al. (6) reported that corneal procurement from LEBJ in 2020 remains unavailable. Meanwhile, as of February 2026, LEBJ documented 234 corneal transplantation waitlists (12). To successfully reduce corneal blindness in Indonesia, local capacity at eye banks to procure corneal tissue must be strengthened. Even though international eye banking networks have reduced inequalities in corneal supply, this should not replace the primary goal of establishing self-sufficient eye banks in every country. Imported tissue should be used only as a temporary solution until national donation issues are resolved (13). In this study, we sought to present corneal donor characteristics from local procurement of LEBJ to better understand Indonesia's local capacity to procure corneal tissue.

## 2 Materials and methods

A consecutive retrospective study of local donor procurement at LEBJ, Indonesia, was conducted from March 2018 to February 2026. This study was approved by the Medical and Health Research Ethics Committee of the Faculty of Medicine, Public Health, and Nursing of Universitas Gadjah Mada–Dr. Sardjito General Hospital (Ref. No: KE/FK/1848/EC/2024) and adhered to the tenets of the Declaration of Helsinki.

There was no age restriction for the donors. The corneal tissues are procured by our certified eye bank technician (CEBT) using the *in situ* corneal excision technique. After the excision, the corneal tissue is stored in a hypothermic medium (e.g., Optisol GS or Cornisol) at 2–8 degrees C. Slit lamp and specular microscopy examinations are performed before the storage and surgery. Routine microbial culture is not performed before the surgery. Corneal tissues were graded based on the ABCD system into Grade A (clear cornea, high ECD ≥ 2,000 cells/mm^2^, use for optical), Grade B (moderate corneal scarring, moderate ECD 1,500–2,000 cells/mm^2^, use for therapeutic), Grade C (significant corneal opacity, poor ECD < 1,500 cells/mm^2^, use for tectonic or patch graft), and Grade D (opaque tissue, severe scaring, non-viable endothelium, use for research and training) according to the EBAA standard (7, 14). The corneal utilization was divided into corneal tissue intended for transplantation and not for transplantation. Routine serology for any infectious screening, including HbsAg, anti-HCV, and anti-HIV, was evaluated. The type of surgery was grouped into superficial anterior lamellar keratoplasty (SALK), superior limbal epithelial transplantation (SLET), keratoprosthesis (KPro), deep anterior lamellar keratoplasty (DALK), penetrating keratoplasty (PKP), and Descemet's stripping automated endothelial keratoplasty (DSAEK). All the procedures in LEBJ follow the AEBA and EBAA medical standards (14, 15).

Corneal donor characteristics, including donor gender, age, cause of death, time from death to procurement, procurement place, ECD and its grades, corneal utilization, type of surgery, and preservation solution, were gathered. The collected data were entered into a Microsoft Excel spreadsheet. We analyzed the data using SPSS Statistics for Mac version 25 (SPSS Inc., Chicago, Illinois, USA). The Shapiro-Wilk test was used to assess the normality of the distribution; our data were not normally distributed. Descriptive statistics were used for continuous variables [mean ± standard deviation (SD)] and categorical variables (frequencies and percentages). Pearson's chi-square test was used to compare baseline characteristics, and Spearman's rank correlation (Spearman's rho) was used to evaluate bivariate analysis. Multivariable binary logistic regression analyses were also performed to identify independent factors associated with Grade A ECD and corneal tissue utilization. Donor age, gender, time of procurement, and preservation solution were included as independent variables. A *P*-value of < 0.05 was considered statistically significant.

## 3 Results

**Figure 1:**
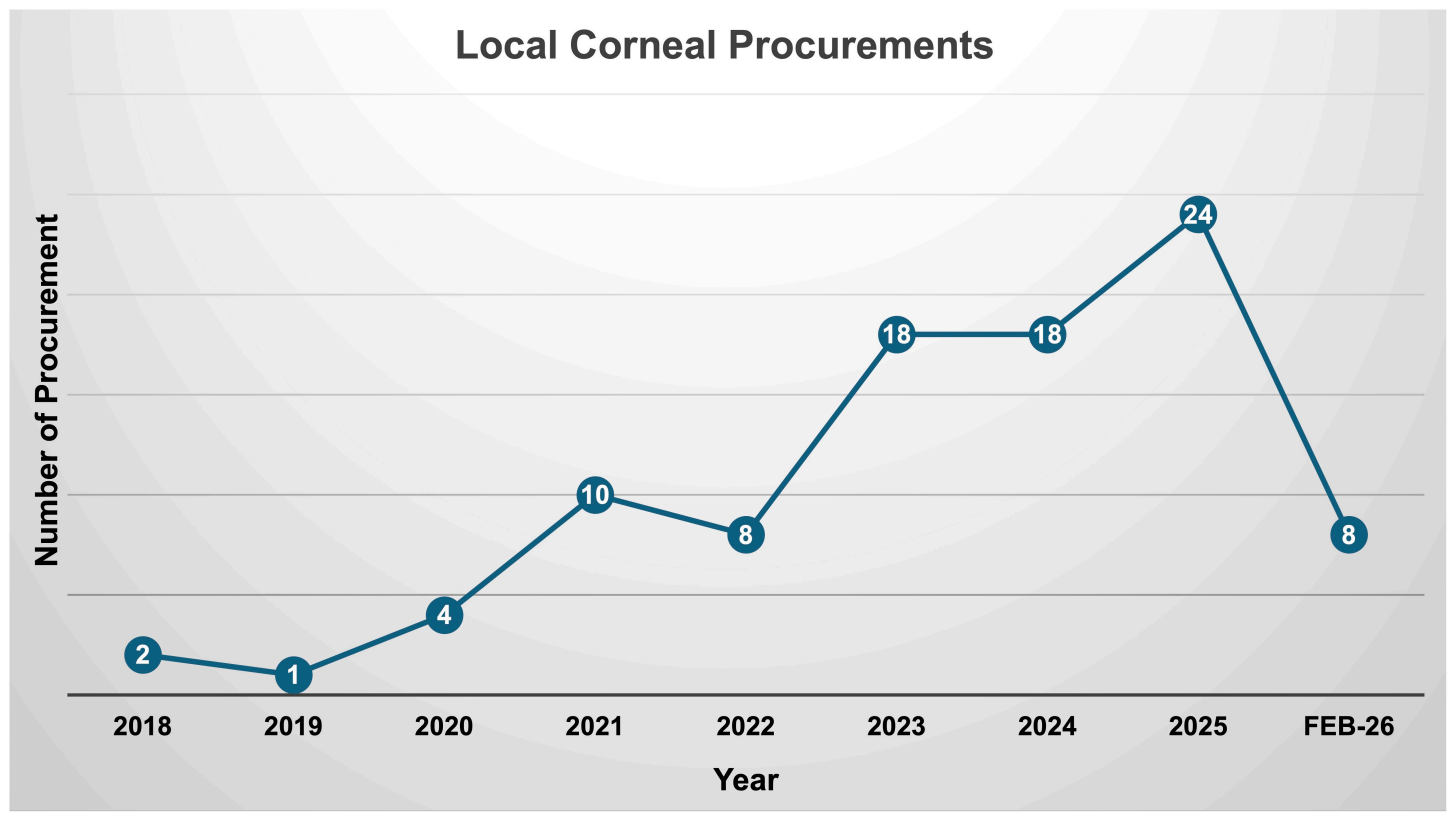
Trends of local corneal procurement.

We documented 49 donors from our local procurement, but two donors' families refused the procedure. Therefore, a total of 47 donors (93 corneal tissues) were included in the study analysis. The average number of local donor procurements at our eye bank was 10.3 corneal tissues per year. From 2018 to 2022, there were unstable trends in the proportion of corneal procurements, but after 2023, procurements gradually increased, reaching a high of 24 (25.8%) corneal tissues in 2025 (Figure 1).

Male donors (24, 51.1%) pre-dominated the cohort. The mean (SD) donor age was 64.60 (19.83) years, with a range of 10 to 100 years. Cardiac arrest (13, 27.7%) was the majority cause of death for the overall cohort, followed by cerebrovascular disease (12, 25.5%). Cardiac arrest was also the leading cause of death among male donors, 2.16 times more than in female donors. Funeral homes (32, 68.1%) were the most common place of procurement. The mean (SD) time from death to procurement was 4.94 (3.04) hours, ranging from one to 16.08 hours. The details on the characteristics of corneal donors are provided in Table 1.

**Table 1:** Donor characteristics from local procurements.

Details	TOTAL (*N* = 47 donors, 100%)
Gender	
Female	23 (48.9%)
Male	24 (51.1%)
Age group (years)	
< 18	1 (2.1%)
18–40	6 (12.8%)
41–65	15 (31.9%)
>65	25 (53.2%)
Cause of death	
Cardiac arrest	13 (27.7%)
Cerebrovascular disease	12 (25.5%)
Lung disease	4 (8.5%)
Cancer	4 (8.5%)
Renal disease	3 (6.4%)
Unknown	2 (4.3%)
Thyroid storm	1 (2.1%)
After major surgery	1 (2.1%)
Procurement place	
Funeral home	32 (68.1%)
Home	9 (19.1%)
Hospital	4 (8.5%)
N/A	2 (4.3%)

A total of 93 corneal tissues were successfully procured. The mean (SD) of ECD was 2,270.03 (308.36) cells/mm^2^, with the majority of corneal tissues documented as Grade A (84, 90.3%). Optisol GS (69, 74.2%) was the most common preservation solution in our cohort (Table 2).

**Table 2:** Corneal tissue characteristics from local procurements.

Details	TOTAL (*N* = 93 tissues, 100%)
ECD grade	
A	84 (90.3%)
B	5 (5.4%)
C	1 (1.1%)
D	0
N/A	3 (3.2%)
Tissue preservation solution	
Optisol GS	69 (74.2%)
Cornisol	24 (25.8%)

**Figure 2:**
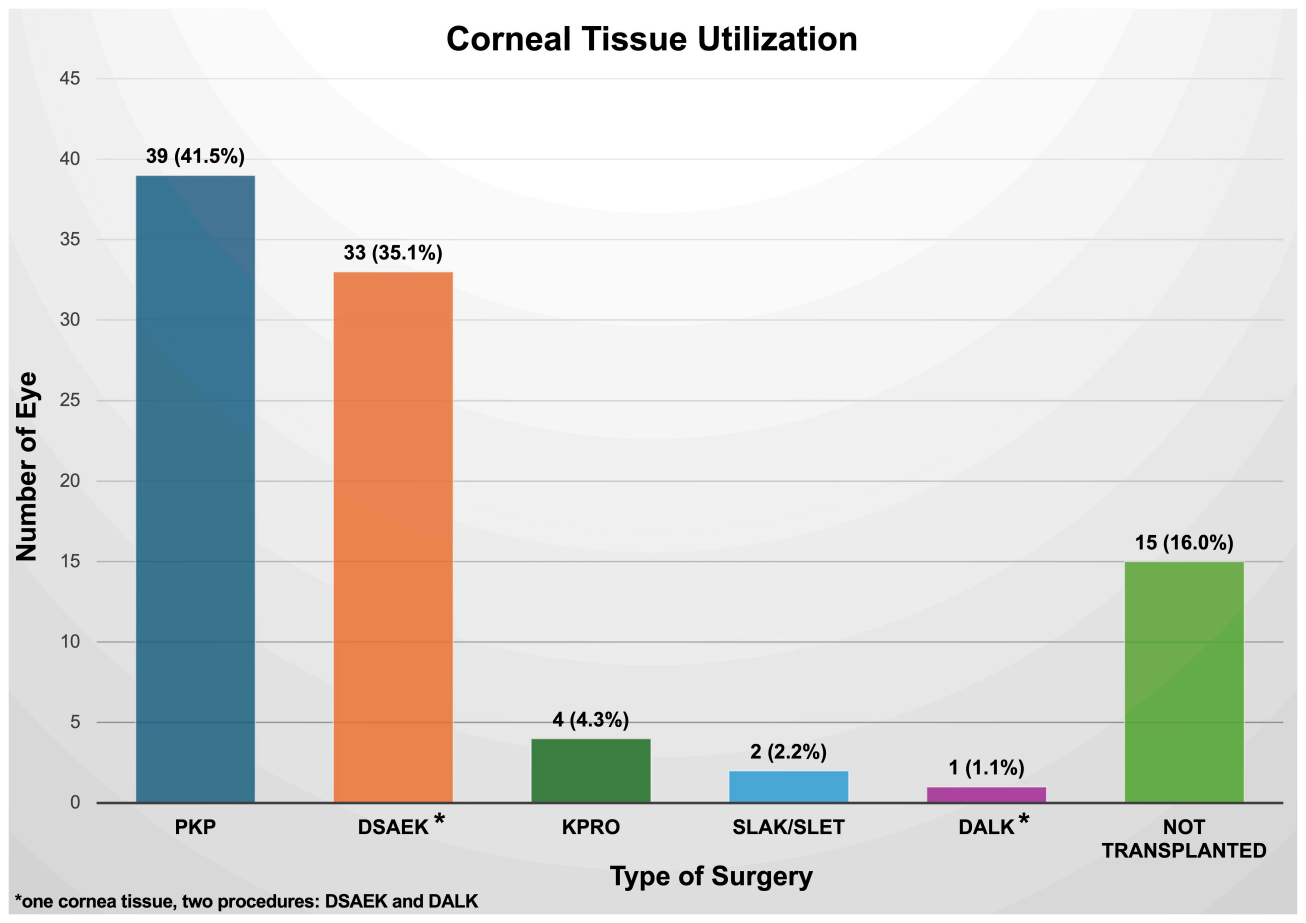
Utilization of corneal tissue from local procurement: PKP, penetrating keratoplasty; DSAEK, Descemet's stripping automated endothelial keratoplasty; KPro, keratoprosthesis; SALK,superficial anterior lamellar keratoplasty; SLET, superior limbal epithelial; DALK, anterior lamellar keratoplasty. ^*^1 cornea tissue, two procedures: DSAEK and DALK.

Of 93 corneal tissues, 81 (87.1%) were intended for transplantation; only 78 corneal tissues were transplanted, with one (1.1%) tissue used for two surgeries (DSAEK and DALK), and three (3.2%) tissues were due to the expiry date being reached; and 12 (12.9%) tissues were not for transplantation, eight (8.6%) due to formalin, three (3.2%) hemolyzed blood sample, one (1.1%) not enough blood sample. We do not perform routine microbiological culture before transplantation. Of the corneal tissues intended for transplantation, we did not find any serology-positive results in the collected blood sample. Therefore, 79 (84.0%) surgeries were performed from 78 corneal tissues (one tissue, two procedures: DSAEK and DALK), and 15 (16.0%) tissues were not transplanted. The mean (SD) time from preservation to surgery was 9.25 (2.8) days, with a range of 1 to 14 days. PKP (39, 41.5%) was the most common surgery, followed by DSAEK (33, 35.1%). Details on corneal tissue utilization are shown in Figure 2.

The Pearson chi-square test demonstrated statistically significant associations between corneal utilization and ECD grades (*P* < 0.001), and between corneal utilization and cause of death (*P* = 0.028). No statistically significant differences were observed between ECD grade and donor gender (*P* = 0.252), age groups (*P* = 0.526), cause of death (*P* = 0.817), or preservation solution (*P* = 0.677).

**Figure 3:**
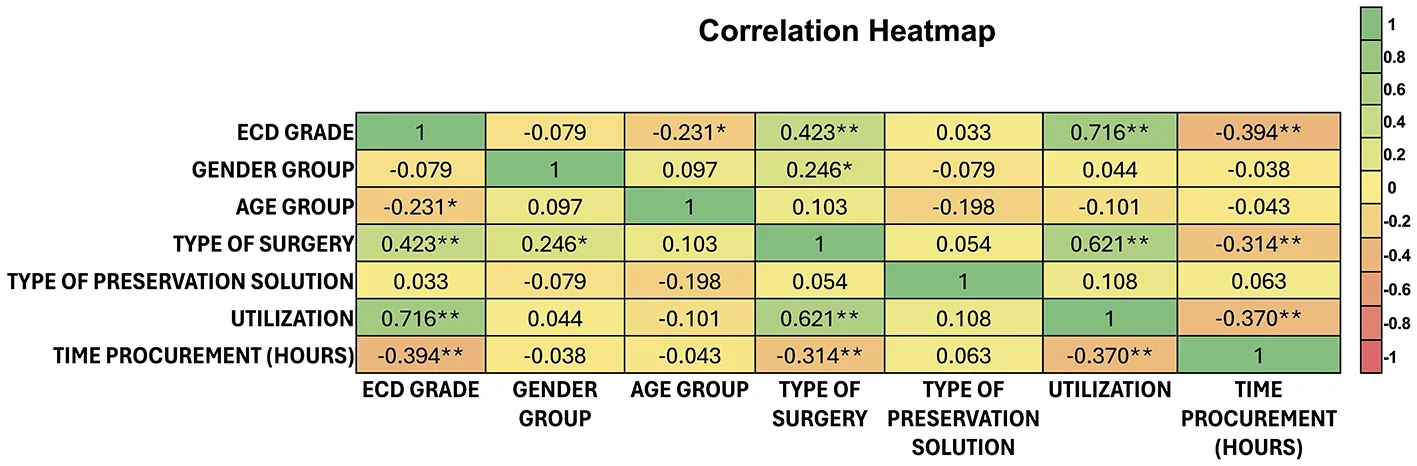
Correlation coefficients between variables using Spearman's rho.

The Shapiro-Wilk test indicated that the data were not normally distributed; therefore, non-parametric analyses were performed. Spearman's rank correlation test showed a weak negative correlation between ECD grades and age group (Spearman's rho; ρ = −0.231, *P* = 0.028) and procurement time (ρ = −0.394, *P* < 0.001). A moderate positive correlation was found between ECD grade and type of surgery (ρ = 0.423, *P* < 0.001), while a strong positive correlation was observed between ECD grade and corneal utilization (ρ = 0.716, *P* < 0.001). Corneal utilization also showed a strong positive correlation with the type of surgery (ρ = 0.621, *P* < 0.001) and a moderate negative correlation with the time of procurement (ρ = −0.370, *P* < 0.001). The Spearman's rho correlation coefficients between variables are provided in Figure 3.

Multivariable analysis using binary logistic regression was performed to assess associations between donor age, gender, time of procurement, and preservation solution, and ECD grade and utilization. No significant association between donor age (OR 0.95; 95% CI 0.87 to 1.032; *P* = 0.225), gender (OR 0.288; 95% CI 0.037 to 2.221; *P* = 0.232), and preservation solution (OR 1.017; 95% CI 0.125 to 8.30; *P* = 0.987) was observed for ECD grade. A longer procurement time was independently associated with reduced odds of obtaining Grade A corneal tissue (OR 0.999; 95% CI 0.998 to 1.000; *P* = 0.025). In contrast, donor age (OR 0.997; 95% CI 0.963 to 1.033; *P* = 0.886), gender (OR 1.213; 95% CI 0.356 to 4.128; *P* = 0.757), and preservation solution (OR 2.391; 95% CI 0.607 to 9.422; *P* = 0.213), and time of procurement (OR 0.999; 95% CI 0.999 to 1.000; *P* = 0.092), were not associated with corneal tissue utilization.

## 4 Discussion

Corneal opacity is currently the fifth leading cause of blindness worldwide (16). The imbalance between clinical demand and corneal donor supply remains a global issue, with almost 13 million on the waitlist for corneal transplantation (7). One of the keys to successful corneal transplantation includes sufficient amounts of eye banking and high-grade corneal tissue. Therefore, assessing the status of local corneal tissue procurement in each country is crucial to at least reduce the number of cases of corneal blindness nationally. Until now, there is still no national registry of corneal transplantation and donors in Indonesia. Even though our study is a small, single-center study, it is the first to report the profile of corneal donors from a local procurement eye bank in Jakarta, Indonesia, comprising 47 donors (93 corneal tissues) over 8 years.

Established in 2018, LEBJ faced very challenging first 3 years, as corneal donation was a new concept in society. Facing challenges in Indonesia, including diverse religious beliefs and misconceptions about corneal donors, LEBJ has actively promoted public awareness and socialized corneal donation. This was reflected in the fluctuating trends that persisted in our cohorts; it was not until 2023 that we observed a significant increase in corneal procurement. These similar challenges are also found in other eye banks (17).

LEBJ started the hospital corneal retrieval program (HCRP) in 2024. However, our data showed that the number of corneal tissues retrieved in hospitals was lower than in the funeral homes. The majority of corneal procurements were voluntary (within the community) and were procured at funeral homes. Hopefully, in the future, HCRP corneal collection will increase, as a previous report showed that HCRP contributed the majority of cases compared to community corneal collection (17, 18).

Male donor preponderance was observed in our cohort, consistent with other reports (17, 19–23). We also noted that cardiac arrest was the leading cause of death in the cohorts, with male donors at 2.16 times higher risk than female donors. Our findings aligned with previous reports from Nepal (17), the United States member of EBAA (20), Western Australia (23), New Zealand (22), and North India (21), except in the international member of EBAA (20), where malignancies were predominant. We have provided detailed findings from other eye bank surveys in Table 3.

**Table 3:** Comparison with other eye banks.

References	Country	Eye bank	Donors (*N*)	Corneal tissue (*N*)	Male donor (%)	Mean age (SD; years)	Majority cause of death (%)	Main procurement site (%)	Mean ECD (SD; cells/mm^2^)	Keratoplasty (%)	Utilization rate (%)
Patel, 2005 (22)	New Zealand	New Zealand National Eye Bank	1,628	3/221	69.8	59.4 (18.3)	Cardiovascular disease (50.5%)	Coroner's service (67.6%)	3,024 (324)	PKP (75.8)	79.4
Bajracharya, 2020 (17)	Nepal	Nepal eye bank	639	1,244	57.7	49.4 (range 5–80)	Cardiovascular disease (27.2%)	HCRP	2,850 (520)	N/A	97
Arya, 2021 (21)	North India	Eye Bank of North India	1,646	851	58.3	63.2 (19.5)	Cardiovascular disease	HCRP (42%)	N/A	PKP (26.5)	56.7
Kezic, 2023 (23)	Western Australia	Lions Eye Bank Western Australia	3,328	6,209	66	57 (16.6)	Cardiovascular disease (29.5%)	HCRP	N/A	PKP (66.4)	84
Van Meter, 2025 (20)	United States	EBAA member	71,778	85,926	60.1	60 (14.6)	Cardiovascular disease (32.7%)	N/A	N/A	EK (48.3)	98.5
Van Meter, 2025 (20)	International	EBAA member	3,237	3,799	59.4	64.7 (12.8)	Cancer (38.6%)	N/A	N/A	EK (56.2)	80.7
Ardiani, 2026 (present study)	Indonesia	Lions Eye Bank Jakarta	47	93	51.1	64.60 (19.83)	Cardiovascular disease (27.7%)	Funeral homes (68.1%)	2,270 (308.36)	PKP (41.5)	84

EBAA, Eye Bank Association of America; ECD, endothelial cell density; EK, endothelial keratoplasty; HCRP, hospital corneal retrieval program; N/A, not available; PKP, penetrating keratoplasty; SD, standard deviations.

LEBJ has no age restrictions for corneal donors. We documented that the donors' ages ranged from 10 to 100 years, with a mean of 64.60 (19.83) years. As reported by Adebayo et al. (19) and Patel et al. (22), donor age is likely not clinically relevant when comparing donors younger than 75 years with those older than 75 years in terms of tissue viability and transplantation suitability.

Even though the majority of corneal tissues were Grade A (84, 90.3%) for optical use, the mean of ECD from corneal tissues in our eye bank was slightly lower compared with other eye banks (17, 22, 24). The reason for these findings could be the older age distribution compared to the previous reports. We found a weak negative correlation between ECD grades and age group (Spearman's rho; ρ = −0.231, *P* = 0.028); older donor age was associated with lower ECD grade. Our findings were aligned with other reports (18, 22). Compared with eye banks in Nepal (17), New Zealand (22), and India (24), our data showed a mean time from death to preservation of 4.94 (3.04) hours, which was shorter. We found a moderate negative correlation between procurement time and ECD grade (ρ = −0.394, *P* < 0.001). The longer the procurement time after death, the lower the ECD. Of 84% of corneal tissues utilized for transplantation, PKP was the most common procedure, accounting for 41.5%, followed by DSAEK (35.1%). These findings remained consistent with our previous keratoplasty trends (8), and were similar to those reported by other eye banks (23). Our data also revealed a strong positive correlation between ECD grade and corneal utilization (ρ = 0.716, *P* < 0.001) and a strong positive correlation between corneal utilization and the type of surgery (ρ = 0.621, *P* < 0.001); the higher the ECD grade, the higher the utilization proportion and the more complex the surgery, including PKP and DSAEK. However, using binary logistic regression, we found that gender, age group, and preservation solution were not associated with ECD grade or utilization. Only the procurement time was associated with ECD grade (OR 0.999; 95% CI 0.998 to 1.000; *P* = 0.025). Longer procurement time after death was associated with lower odds of obtaining Grade A corneal tissue for optical use.

Of the 93 corneal tissues, 12 (12.9%) were not suitable for transplantation; eight (8.6%) were due to formalin; three (3.2%) were hemolyzed blood samples; and one (1.1%) had insufficient blood. The formalin contamination, hemolyzed blood samples, and insufficient blood samples prevented serological testing. We could not know the serology status regarding HIV, HBV, and HCV. Hence, they are categorized as not for transplanted tissues. This category of non-transplantable tissue is not based on microbiological culture results. We do not perform routine microbiological culture before transplantation in our eye bank, since bacteriologic contamination of donor eyes typically does not lead to infection;(25) however, in accordance with AEBA and EBAA medical standards, it may be performed, and is not mandatory (14, 15). Meanwhile, the other surveys revealed that 16% of tissues were not transplanted due to formalin fixation, hemolysis, and insufficient blood samples. Our data showed a lower rate of non-transplant tissues than in other eye bank reports (17, 23).

We noted that three (3.2%) corneal tissues reached the expiry date in our cohort, despite 234 waitlists at our eye bank. The high cost of corneal transplantation in Indonesia is due to Indonesia's national insurance system, which does not cover the cost of corneal tissue processing. Therefore, the costs of corneal transplantation could not be afforded by all waitlisted patients. Additionally, most of our waitlisted patients are from across Indonesia and outside of Jakarta; challenges in matching patient schedules with surgeon availability and tissue expiration windows also contributed to patient readiness. These factors accounted for our corneal tissue reaching its expiry date.

In a 2021 editorial article by Jeng and Ahmad (13), the authors argued that to reduce the number of cases of corneal blindness worldwide, leaders and governance need to understand and recognize the need for corneal transplantation in their countries. Because active government involvement also plays an important role in facilitating a successful eye banking infrastructure. According to the Indonesian Ophthalmology Association, one in 1,000 patients has corneal blindness. To successfully reduce corneal blindness in Indonesia, the active involvement of government stakeholders should be strengthened, including support from the Indonesian national insurance, not only for corneal transplantation but also for the cost of corneal processing. Therefore, waitlisted patients can afford the procedure, and hopefully, there is no other waitlist for corneal transplantation in Indonesia.

Although during the study period, LEBJ underwent progressive development in organization, including affiliation with Sightlife (2018), membership in AEBA (2020) and GAEBA (2024), implementation of HCRP (2024), and standard certification of the Ministry of Health of the Republic of Indonesia (2025), our eye bank protocols remain consistent with AEBA and EBAA medical standards (14, 15).

Our study has some limitations, including incomplete data recorded in the first year our eye bank was established. Even though our study was conducted at a single eye bank in Indonesia and the number of corneal procurements in this cohort remains small compared with other eye banks, our data reflect the real situation in Indonesia, where there is a lack of corneal donation and very poor eye banking infrastructure. There may also have been possible selection bias in this study and limited statistical power due to its single-center design. The Indonesian national registry for corneal transplantation and donor tissue remains unavailable and is urgently needed. Furthermore, since corneal donation is a new concept in our country, some challenges persist, including diverse religions and beliefs, as well as misconceptions and myths about corneal donors and the HCRP implementation. Despite LEBJ already having HCRP collaborations, willingness and approval to donate their family's corneas remain low because the new concept of corneal donation in Indonesia differs from that in other countries, where organ donation, including the cornea, is well implemented (26–29). We expect more corneal collections at the hospital with which we collaborated. As reported by Sharma et al. (30), HCRP corneal collections can reduce the demand-supply deficit in corneal transplantation. Therefore, active involvement between the Indonesian government and existing eye banks is vital. The recent increase in our corneal procurement may reflect the impact of our community awareness initiatives, although other organizational developments during the study period may also have contributed. However, procurement sites were mainly obtained voluntarily from funeral homes; they may not represent all deaths in Indonesia, including underrepresentation of rural populations, since our eye bank is located in Jakarta, Indonesia, and cultural differences may also influence the findings in donor profiles.

In conclusion, male donors, older age, and cardiac arrest were the most common donor characteristics. Funeral homes were the primary place for corneal procurement. Most corneal tissues were Grade A for optical use, with PKP being the most common surgery. Gender, age group, and preservation solution were not independently associated with the ECD grade and utilization. Procurement time was the only independent predictor of obtaining Grade A corneal tissue; longer procurement time after death was associated with reduced odds of obtaining Grade A corneal tissue for optical use. However, given the small sample sizes from a single eye bank in Jakarta, Indonesia, these findings should be interpreted with caution.

## Data Availability

The datasets presented in this article are not readily available because of confidentiality reasons, but are available from the corresponding author on reasonable request. Requests to access the datasets should be directed to Sharita R. Siregar, sharita@jec.co.id.
